
JOURNAL INFORMATION
==============================
NLM Title Abbreviation: ScientificWorldJournal
Journal ID: ScientificWorldJournal
NLM Journal ID: 101131163
Journal ID: TSWJ

The Scientific World Journal

ISSN: 2356-6140
EISSN: 1537-744X
Publisher: Wiley

ARTICLE INFORMATION
==============================
PMCID: PMC6084164
PMID: 30147588
DOI: 10.1100/tsw.2001.121
Article ID: 490385
Article version: 1
Subjects: Short Report

Pan-Caspase Inhibitor Prevents Anti-CD3 Mediated T-Lymphocyte Proliferation

Mahidhara Raja S. *
Hoffman R.
Simmons R.L.
Billiar T.R.
Department of Surgery, University of Pittsburgh School of Medicine, W1503 Biomedical Science Tower, 200 Lothrop St., Pittsburgh, PA 15261, USA
*Raja S. Mahidhara: mahidharar@msx.upmc.edu
Publication date: 2001
Electronic publication date: 2001 Dec 12
Volume: 1
First page: 107
Last page: 107
Copyright: Copyright © 2001 Raja S. Mahidhara et al.
Copyright year: 2001
License: This is an open access article distributed under the Creative Commons Attribution License, which permits unrestricted use, distribution, and reproduction in any medium, provided the original work is properly cited.
License URL: https://creativecommons.org/licenses/by/3.0/

Funding: National Institutes of Health RO1-GM-44100

==============================
Miami *Nature Biotechnology* Short Reports
*TheScientificWorld* (2001) 1(S3), 107SR
ISSN 1532-2246; DOI 10.1100/TSW.2001.121

### PAN-CASPASE INHIBITOR PREVENTS ANTI-CD3 MEDIATED T-LYMPHOCYTE PROLIFERATION

Raja S. Mahidhara*, R. Hoffman, R.L. Simmons, and T.R. Billiar

Department of Surgery, University of Pittsburgh School of Medicine, W1503 Biomedical Science Tower, 200 Lothrop St., Pittsburgh, PA 15261
* mahidharar@msx.upmc.edu

**BACKGROUND.** Host defense requires the ability for upregulation and downregulation of lymphocyte responses. It is well known that proliferative responses can be seen in conjunction with subsequent cell death suggesting that the two processes are linked.(1) Caspases are constitutively expressed zymogens which, upon activation, participate in receptor and non-receptor mediated apoptosis.(2) The purpose of this study was to assess whether caspase activity was required for the proliferation of splenic-derived T lymphocytes by using the non-specific, irreversible inhibitor z-VAD-fmk.

**METHODS.** Splenocytes from C57Bl/6 mice were passed over an antibody coated column (R&D) to obtain a >90% CD3+ population. Cells were stimulated to proliferate with immobilized monoclonal anti-CD3 (BD Pharmingen, 100µg/ml) in 10% MLC media. Proliferation was assessed by $^{3}$H thymidine incorporation at time points with and without z-VAD-fmk (Alexis, 100µM). Western Blot analysis was performed by SDS-PAGE and probed with polyclonal Caspase 3 antibody H-277 (Santa Cruz, 1:1000). Caspase 3-like activity was assessed by 100µM Ac-DEVD-pNA (Alexis) substrate followed by quantitation by spectrophotometry at 405nm.

**RESULTS.** TCR stimulation of purified T lymphocytes at 72 hours resulted in proliferation 500 fold greater than unstimulated controls. Treatment of lymphocytes with zVAD inhibited CD3 mediated proliferation by greater than 99%. Delay in the administration of inhibitor after stimulation decreased the effect on proliferation (see Figure 1).

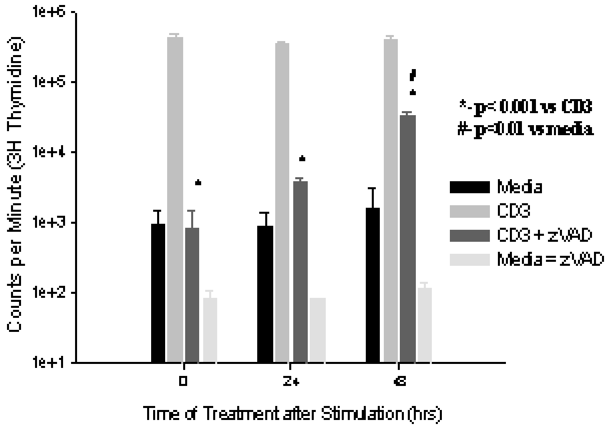

Western blot revealed the appearance of the 17kD active form of caspase 3 after 48 hours of stimulation (data not shown). Caspase 3-like activity was similarly seen to increase over time after stimulation to greater than 3-fold over control by 72 hrs. Change in OD/mg/hr went from 2.0 at time 0 to 4.8 at 24 hours and 6.3 by 72 hrs (Figure 2).

**Figure 2. DEVD like activity in CD3 stimulated Naive T cells**

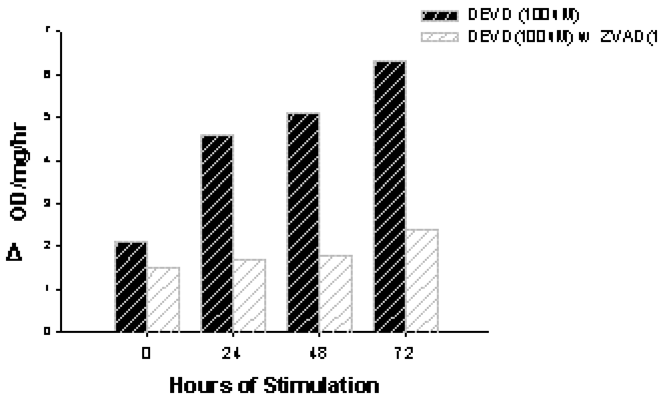

**DISCUSSION.** Experiments in this study have shown that the non-specific caspase inhibitor, z-VAD, inhibits polyclonal stimulation of naïve, murine spleen derived T lymphocytes. Stimulation of lymphocytes is associated with time dependent expression of the active fragment of caspase 3, a central effector molecule in the apoptotic pathway. Importantly function of caspase 3 was observed by enzyme activity and seen to increase in a time dependent manner with stimulation.

Members of the caspase family of cysteine proteases have been known to be intimately involved with a variety of essential steps in lymphocyte apoptosis. We show that these molecules may also be involved with proliferative responses. Regulation of caspase signaling may be an important link in understanding the link between proliferation and cell death after T cell stimulation.

**ACKNOWLEDGEMENTS**. This work was supported by Society of Thoracic Surgeons Research Fellowship (RSM) and the NIH RO1-GM-44100 (TRB).

**REFERENCES.**

1. Lenardo, M. et al. (1999) Annu. Rev. Immunol. 17, 221-253
2. Budihardjo, I. et al. (1999) Annu. Rev. Cell Dev. Biol. 15, 269-290
